# Explaining Attitudes and Adherence to Antipsychotic Medication: The Development of a Process Model

**DOI:** 10.1155/2014/341545

**Published:** 2014-02-19

**Authors:** Martin Wiesjahn, Esther Jung, Fabian Lamster, Winfried Rief, Tania M. Lincoln

**Affiliations:** ^1^Department of Psychology, Division of Clinical Psychology and Psychotherapy, Philipps-Universität Marburg, 35032 Marburg, Germany; ^2^Institute of Psychology, Department of Clinical Psychology and Psychotherapy, Universität Hamburg, 20146 Hamburg, Germany

## Abstract

Although nonadherence to antipsychotic medication poses a threat to outcome of medical treatment, the processes preceding the intake behavior have not been investigated sufficiently. This study tests a process model of medication adherence derived from the Health Belief Model which is based on cost-benefit considerations. The model includes an extensive set of potential predictors for medication attitudes and uses these attitudes as a predictor for medication adherence. We conducted an online study of 84 participants with a self-reported psychotic disorder and performed a path analysis. More insight into the need for treatment, a higher attribution of the symptoms to a mental disorder, experience of less negative side effects, presence of biological causal beliefs, and less endorsement of psychological causal beliefs were significant predictors of more positive attitudes towards medication. The results largely supported the postulated process model. Mental health professionals should consider attitudes towards medication and the identified predictors when they address adherence problems with the patient in a shared and informed decision process.

## 1. Introduction

A substantial proportion of patients with psychotic disorders do not take their medication as prescribed. Estimations of the frequency of nonadherence to antipsychotic medication range widely depending on the definition of adherence. The nonadherence rate is estimated to be 49.5% based on a definition of adherence as “taking medications as prescribed at least 75% of the time” [1, page 901]. Nonadherence can be problematic as medication withdrawal has been found to be associated with a higher risk of relapse [2], increased hospital admission rates [3], and in turn high costs for the health care system [4]. Research on dopaminergic supersensitivity [5, 6] indicates that in certain cases irregular intake and sudden dose reductions may be worse than taking no medication at all. Moncrieff [5] concludes that in some patients relapse into psychosis “may be a feature of drug withdrawal rather than the re-emergence of an underlying illness” (page 3).

In order to address the risks associated with sudden discontinuation of antipsychotic medication it is essential to understand the processes that lead to negative attitudes towards the medication and to nonadherence. For this purpose it appears promising to evaluate the individual costs and benefits of antipsychotic medication for each individual patient as described in the Health Belief Model (HBM [7, 8]) which was developed to explain general health behavior by evaluative processes. In the course of a shared decision process [9, 10] the clinician needs to inform the patient about potential benefits and unwanted effects of medication and the risk associated with nonadherence. Beyond weighing the pro of likely effectiveness and the con of side effects, the patient's adherence and attitudes towards medication are likely to be affected by previous experiences, social influences, or even symptomatology in itself. Knowing the relevant factors that explain attitudes and adherence can help clinicians to support patients in arriving at an informed decision about the treatment options. Such knowledge might also help to further develop interventions aimed at improving adherence [11, 12] for those patients who are likely to benefit from medication.

Previous research has focused on several factors that might be related to medication attitudes and adherence in patients with psychotic disorders. More positive attitudes about medication and medication adherence have been consistently found to be associated with the “insight” into the presence of a mental disorder [1, 13–16] and with a good relationship to the treating physician [1, 14, 17–19]. In Lacro and colleagues' review [1] most studies did not show an association between self-rated side effects and adherence which the authors attempted to explain with the lack of systematic side effect ratings. In support of this explanation, recent studies in patients with psychotic disorders [20, 21] and in other populations [22] applied standardized assessments of side effects and found them to be consistently related to lower medication adherence. The findings on psychotic symptoms as a predictor for medication attitudes and adherence are heterogeneous. In the review by Lacro et al. [1] half of the studies revealed fewer symptoms to be associated with higher adherence, whereas the other half did not find such a relationship. Positive beliefs about symptoms (e.g., “during psychosis, I had a feeling of importance and power” [20, page 3]) were only recently included in the field of research and the study by Moritz et al. [20] indicated their importance for medication adherence. Social support was associated with better adherence in the studies by Coldham et al. [15] and Dassa et al. [19], whereas Lacro and colleagues' review found mixed results in regard to this relationship [1]. One recent study indicates that the attitudes towards medication held by the immediate social environment of the person can also be a reason for discontinuation of medication [16]. The potential impact of causal beliefs about the disorder on medication attitudes and adherence has not been investigated in people with psychotic disorders so far. However, in an experimental study in a healthy sample, a biological causal model increased the motivation to take medication [23].

Following these findings, the relevance of some predictors, such as insight and alliance, seems to be evident. For others further clarification is needed (e.g., social support, attitudes of the immediate social environment, or causal beliefs). Also, this field of research faces several methodological obstacles which make it difficult to interpret and compare the findings. For one, studies have typically used outcome measures that mix the constructs of adherence, attitudes, and side effects, such as the Drug Attitude Inventory [24]. This is problematic as it confounds adherence as the actual behavior with preceding conditions. To understand the processes involved in medication adherence it would be helpful to differentiate between adherence as the behavioral component, attitudes as the evaluative component, and side effects and other “costs and benefits” of the treatment. The HBM [7, 8] could serve as a conceptual framework for this differentiation. Following the HBM, medication adherence is likely to be influenced by subjective attitudes towards the medication, which in turn depend on cost-benefit considerations [8]. As another limitation previous studies mostly included small subsets of possible predictors [14, 15] rather than considering a comprehensive set of predictors. This makes it difficult to estimate the incremental amount of variance explained by each predictor. Furthermore, many studies included only participants who were in psychiatric inpatient treatment at that time which limits the generalizability of the findings. Also, the risk of biased answers could be higher in inpatient settings because patients might fear negative consequences of reported nonadherence.

**Figure 1:**
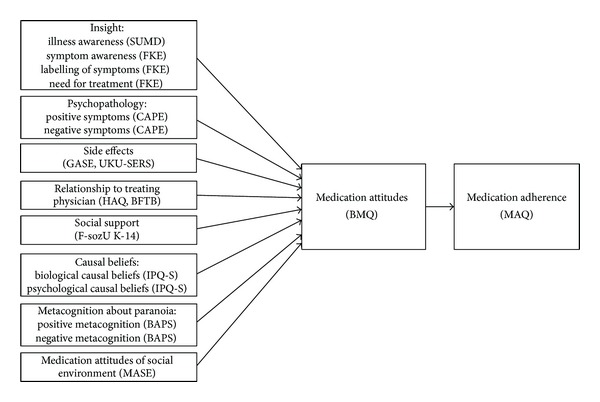
Process model to explain the variance in medication adherence with medication attitudes and possible predictors.

The aim of the present study was to increase our understanding of the processes that are involved in the formation of attitudes towards medication and might impact on medication adherence. With a differentiated and systematic assessment of possible predictors, attitudes towards medication, and medication adherence we investigated a process model derived from the HBM. It integrates an extensive set of possible predictors for attitudes towards antipsychotic medication and uses these attitudes to explain the variance in medication adherence (see Figure 1). We included the set of known predictors (different aspects of insight, positive and negative symptoms, side effects, relationship to the treating physician, and social support) as well as less frequently investigated factors (biological and psychological causal beliefs about the disorder, positive and negative beliefs about paranoia, and the attitudes towards medication held by the immediate social environment). We used an online assessment to reduce selection effects and increase the likelihood of unbiased responses.

## 2. Method

### 2.1. Procedure

The data was collected using SoSci-Survey (https://www.soscisurvey.de/). We invited members of nonprofit online forums on psychotic disorders in German language to participate. The link was also placed in newsletters and notice boards of self-help groups and in public places such as supermarkets in several German cities (a list of all websites and organizations can be retrieved from the first author). The objective of the study was explained (investigation of attitudes and adherence to antipsychotic medication). Participants had to agree to the terms of participation explained on the first page of the study. The completion of the questionnaire took approximately 30 min. As an incentive, a lottery for five 10€ online gift certificates was performed. We included participants who were 18 years or older, completed the full questionnaire, and reported to have been diagnosed with at least one of the following psychotic disorders: schizophrenia, schizoaffective disorder, delusional disorder, or unspecified psychotic disorder. In order to focus on the long term process of medication attitudes and adherence we excluded individuals who reported to have been diagnosed with a brief psychotic disorder. Furthermore, we included only those participants who reported to take antipsychotic medication in the present or have taken it in the past.

### 2.2. Measures

To assess the adherence to antipsychotic medication we used the Medication Adherence Questionnaire (MAQ [25]). The 4-item scale is behaviorally formulated and has been shown to predict actual intake behavior [26]. In previous research the MAQ items showed an internal consistency of *α* = 0.67 [26]. Lüllmann and Lincoln [27] developed an authorized German version by translating and blindly retranslating the questionnaire. The participants were asked to refer all questions to antipsychotic medication only. A higher score indicates better adherence.

To assess attitudes towards antipsychotic medication we used the German version [28] of the Beliefs about Medicines Questionnaire (BMQ [29]). The 18-item instrument refers to positive and negative beliefs about medication. In previous research [28] the BMQ showed a three-factor structure with internal consistencies between *α* = 0.70 and 0.85. We instructed the participants to refer all items to antipsychotic medication. In order to estimate overall attitudes towards antipsychotic medication we calculated the mean score. A higher score implies more positive attitudes.

We assessed the general awareness of having a mental disorder with the first item of the Scale to Assess Unawareness of Mental Disorder (SUMD [30]), which is highly correlated with other general insight measures [13]. The SUMD and its German version are well-established and validated [28]. In the original semistructured interview, items are rated by the interviewer following anchoring criteria. For the purpose of this study, we transformed the first item into a self-report multiple choice item with three response options analogous to the original anchors: “I believe that I have a mental disorder,” “I am not sure that I have a mental disorder but I can entertain the idea that I might,” and “I do not believe that I have a mental disorder.” A higher score indicates more general insight.

For a more differentiated assessment of insight we used the FKE (Fragebogen zur Krankheitseinsicht (Questionnaire of Illness Insight [31])). This German instrument includes the subscales symptom awareness (3 items), labeling of symptoms as a mental disorder (3 items), and insight into the need for treatment (4 items). Internal consistencies were acceptable to good in previous research (*α* = 0.70 − 0.85 [31]). In each subscale higher scores indicate more insight.

We assessed psychotic symptoms with the Community Assessment of Psychic Experiences (CAPE [32]). The CAPE consists of 42 items on the three dimensions: positive symptoms, negative symptoms, and depression. The German version has demonstrated good internal consistencies (*α* = 0.84 − 0.91 [33]). In order to assess specific psychotic symptoms we used the subscales positive symptoms (20 items) and negative symptoms (14 items) with higher scores indicating more symptoms. For reasons of economy we omitted the depression subscale.

To assess medication side effects we used the Generic Assessment of Side Effects (GASE) by Rief et al. [34] who found a high internal consistency of the German version (*α* = 0.89). Participants are instructed to rate the intensity of the presented potential side effects on 4-point scales. In our study the participants were asked whether they attribute the phenomena to antipsychotic medication or not. In order to also assess side effects that are more specific for antipsychotic medication (e.g., “increased salivation”) we added nine items from the UKU Side Effect Rating Scale (UKU-SERS [35]), which assesses side effects of antipsychotic medication. We used the German version [36] and adapted the rating format to the items of the GASE. For the estimation of side effects we used the sum-score of all GASE and UKU-SERS items that were attributed to antipsychotic medication with a higher score implying more side effects.

We assessed the quality of the alliance between the participant and the treating physician with the validated German version of the Health Alliance Questionnaire (HAQ [37]). We used the 6-item HAQ subscale referring to the patient's satisfaction with the therapeutic relationship (*α* = 0.89 [37]). To assess the relationship in more detail we added items from the BFTB (Bonner Fragebogen für Therapie und Beratung (Bonn Questionnaire for Therapy and Consulting [38])). This German instrument contains three relationship scales based on Roger's conceptualization of alliance [39]. Participants are asked as how empathic and genuine they perceive or perceived their treating physician and how accepted they feel or felt. We used a subset of five items for each of the three subscales, which demonstrated excellent internal consistencies in previous research (*α* = 0.90 − 0.91 [38]). The combined assessment of the alliance consisted of 21 items. Participants were instructed to refer the statements to the current or recent treating physician who prescribed the antipsychotic medication. A higher score implies better alliance.

Social support was assessed with the F-sozU K-14 (Fragebogen zur sozialen Unterstützung, Kurzform (Social Support Questionnaire, Short Version [40])). This 14-item instrument assesses the subjective perception of the availability of social support. The instrument has demonstrated excellent internal consistency in previous research (*α* = 0.94 [40]). A higher score indicates more perceived social support.

We assessed the beliefs about the causes of the disorder with items from the German version [41] of the Illness Perception Questionnaire for Schizophrenia (IPQ-S [42]) which assesses a set of beliefs about schizophrenia. Regarding causal beliefs participants are asked to rate their agreement to potential causes on 5-point scales. We used the 13-item psychological and the 4-item biological factors which have shown good to acceptable internal consistencies in previous research (*α* = 0.89 − 0.63 [23]). We used the mean scores of the subscales with higher values indicating higher agreement.

Positive and negative beliefs about paranoid experiences were assessed with the Beliefs about Paranoia Scale (BAPS [43]) which refers to metacognitive beliefs about paranoia. The authors found good to excellent internal consistencies for the subscales survival beliefs, negative beliefs, and normalizing beliefs (*α* = 0.85 − 0.91). Via translation and blinded retranslation we developed a German version, which was approved by the first author of the scale. For the purpose of this study, we included the subscales negative beliefs and, as an indicator of positive metacognition, survival beliefs. Higher scores indicate higher beliefs. The questionnaire was only administered to those participants who reported to have had paranoid experiences.

In order to assess the attitudes of the immediate social environment towards antipsychotic medication we developed a 6-item instrument called Medication Attitude of the Social Environment (MASE). It assesses the perceived opinion of relevant others (“People who are important for me think that it is right that I take antipsychotics.”). The items are to be rated on 5-point scales. In our study the internal consistency of this scale was good (*α* = 0.88). A higher score indicates a more positive attitude.

An overview of the instruments is depicted in Figure 1. Demographic data and clinical history were assessed via self-report.

### 2.3. Analysis

The data was analyzed with SPSS 21 and AMOS 19. In a first step we investigated the bivariate relationships between the variables. This step involved testing whether the variables were normally distributed (Kolmogorov-Smirnov test) to decide whether to use Pearson's (*r*) or Spearman's correlations (*r*_*s*_). Then we calculated correlations between the potential predictors, medication attitudes, and adherence. Additionally, we examined the path model depicted in Figure 1. In the first step we included all possible predictors for medication attitudes into a path analysis (based on a maximum likelihood estimation). Then, we adjusted the path model by reducing the number of predictors in a step by step approach. In each step we excluded the predictor with the lowest path coefficient. Then we reevaluated the path model and excluded the next predictor until only significant predictors were included. Post hoc we tested for possible mediation effects with the Sobel test [44].

## 3. Results

### 3.1. Sample Characteristics

Of the 214 people who accepted the terms of participation and started the assessment, 107 completed the full questionnaire. Twenty-one participants were excluded because they did not report to have been diagnosed with one of the psychotic disorders described in the inclusion criteria. Two individuals were excluded because they reported to have never taken antipsychotic medication. The excluded participants did not differ from the included sample with regard to gender, age, or education. The remaining sample included *n* = 84 participants (54.8% female) with a mean age of 38.3 (SD = 9.7). One quarter (23.8%) were married or in a relationship, 66.7% were single, and 9.5% were divorced. Asked for their level of education, 29.8% reported to have completed university, 36.9% high school, 26.2% intermediate secondary school, and 7.1% general secondary school or below. One third (33.3%) were working on the “first labor market,” 29.8% were not working because they were considered as “disabled due to disorder,” 16.7% were in training, 10.7% were unemployed, 2.4% worked in sheltered workshops, and 7.1% provided no data on employment status.

Regarding diagnoses, 73.8% of the participants indicated to have been diagnosed with schizophrenia, 35.7% with schizoaffective disorder, 8.3% with delusional disorder, and 8.3% with unspecified psychotic disorder. The majority (72.6%) reported one diagnosis, 21.4% two, and 6.0% three or four diagnoses. The mean number of psychotic episodes was 5.8 (SD = 6.8). All participants reported to have received some kind of treatment for their mental health problems; 89.3% were currently in treatment and 10.7% had received treatment in the past. The majority (92.9%) had been admitted to a psychiatric clinic in the past. Most participants (81.0%) were taking antipsychotic medication at the time of data collection and 19% indicated that they had been taking medication in the past but not now.

### 3.2. Bivariate Relationships between Medication Attitudes and Predictors

The bivariate analyses (see Table 1) showed a positive correlation between medication attitudes and medication adherence (*r*_*s*_ = 0.30,  *P* < 0.01). We found significant positive correlations between attitudes towards medication and the four components of insight (illness awareness, *r*_*s*_ = 0.23,  *P* < 0.05; symptom awareness, *r* = 0.24,  *P* < 0.05; labeling of symptoms as a mental disorder, *r* = 0.34,  *P* < 0.01; and insight into the need for treatment, *r* = 0.53,  *P* < 0.001). Also, more positive attitudes towards medication were associated with fewer side effects (*r* = −0.36,  *P* < 0.01), a better relationship to the treating physician (*r* = 0.34,  *P* < 0.01), higher biological causal beliefs (*r* = 0.43,  *P* < 0.001), more negative metacognition about paranoia (*r* = 0.31,  *P* < 0.01), and better medication attitudes of the immediate social environment (*r* = 0.38,  *P* < 0.001).

### 3.3. Results from the Path Analysis

**Figure 2:**
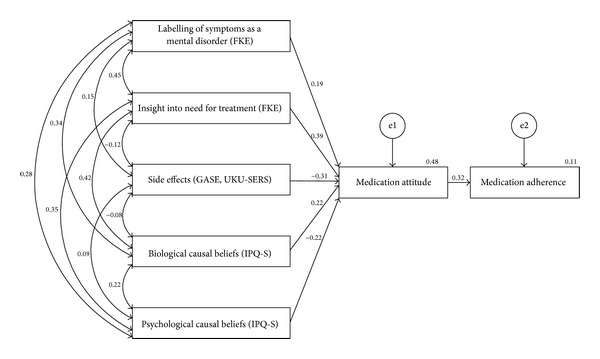
Path analysis to explain the variance in medication adherence with medication attitudes and related variables.

We included all 14 possible predictors for medication attitudes in the first step of the path analysis. In nine steps we reduced the number of predictors until only significant predictors remained in the model. The final model (compare Figure 2) contained five predictors for more positive attitudes towards medication: higher scores in the scale labeling the symptoms as a mental disorder (*β* = 0.19;  *P* < 0.05), more insight into the need for treatment (*β* = 0.39;  *P* < 0.001), fewer side effects (*β* = −0.31;  *P* < 0.001), more biological causal beliefs (*β* = 0.22;  *P* < 0.05), and fewer psychological causal beliefs (*β* = −0.22;  *P* < 0.01). Forty-eight percent of the variance in medication attitudes and eleven percent of the variance in medication adherence were explained.

We considered several aspects of model fit. The nonsignificant *χ* ^2^  (*χ* ^2^ = 4.29,  df = 5,  *n*.*s*.) is a first indicator for good fit. The Normed Fit Index (NFI) compares the *χ* ^2^ of the model to the *χ* ^2^ of the independence model. The score of 0.97 is above the cutoff 0.95 indicating a good fit. The Goodness of Fit Index (GFI), which refers to the proportion of variance and covariance explained by the model, has a cut-off score of 0.95 and also indicates a good fit for our data (GFI = 0.99). The Comparative Fit Index (CFI) describes the incremental power of the model in comparison to the independence model. The score of 1.00 was clearly above the cut-off score of 0.95 which also indicates a good fit. The overall fit index RMSEA (Root Mean Square Error of Approximation) compares the proposed model to the saturated model with smaller values indicating a better fit. In our data the RMSEA was set at 0.00 because the *χ* ^2^ score was lower than the number of degrees of freedom (df). Although this value indicates a close fit [45], the 90% confidence interval for the RMSEA is relatively wide (from 0.00 to 0.14). This indicates an imprecision of the estimation which might be due to the relatively small sample size [46].

## 4. Discussion

Our findings largely support the postulated process model, which emphasizes the importance of individual evaluative processes that precede medication intake behavior. According to our path analysis, fewer perceived side effects, a higher attribution of the symptoms to a mental disorder, a greater sense of needing treatment, more endorsement of biological causes of the disorder, and less approval to psychological causes were associated with more positive attitudes towards medication, which in turn partly predicted better adherence.

### 4.1. Predictors for Attitudes towards Antipsychotic Medication

The association between more positive attitudes towards medication and higher scores in the two facets of insight (labeling the symptoms as a mental disorder and insight into the need for treatment) is in line with previous research [1, 13–16]. However, our results go beyond earlier findings as we assessed various aspects of insight and controlled for intercorrelations between the potential predictors. We identified labeling of the symptoms and insight into need for treatment to be the only aspects of insight that explained an incremental amount of variance in medication attitudes. A possible explanation for the specific importance of these facets is that they evaluate the person's own situation (“It is good for my health to have regular contact to a psychiatrist.”) and symptoms (“Because of a mental disorder I have unusual experiences that occur only in my head.”). Thus, in order to see the benefits of the medication it seems to be important that the person perceives his or her individual symptoms as being a part of some kind of disorder that needs to be treated.

We assessed side effects in a systematic and extensive manner and found more side effects to be associated with more negative medication attitudes. This is in line with recent research [20–22] and supports the hypothesis that earlier null findings underestimated the relevance of side effects for medication attitudes due to insufficient measurement methods [1].

The relevance of biological causal beliefs to medication attitudes indicates that it is important that the person perceives the treatment as being plausible with regard to the etiology of the disorder. For individuals who believe their disorder to have biological causes it probably “makes sense” and appears to be beneficial to accept a biological treatment, that is, medication.

In contrast, the results of the path analysis suggest that psychological causal beliefs antagonize the establishment of positive medication attitudes. According to this finding a stronger belief in psychological causes might question the perceived benefits of antipsychotic medication. This would partly contradict the results by Lüllmann et al. [23], who found that clinicians should aim at an etiological model that combines psychosocial and biological causes because this combination fosters the success of various kinds of treatment, including medication. However, in our data psychological causal beliefs received a significant path coefficient (*β* = −0.22;  *P* < 0.01) although the variable was not significantly correlated to medication attitudes in the bivariate analysis (*r* = −0.01, *n.s.*). This indicates a suppression effect; that is, one or more of the other predictors in the path analysis suppress irrelevant variance in the variable and thereby statistically increase its predictive power. We performed post hoc analyses and separately combined psychological causal beliefs with each of the other predictors. Labeling of symptoms was revealed to be the only suppressor variable; that is, psychological causal beliefs can only be incorporated into the model if labeling of symptoms is included. This restricts the distinct importance of the variable and thereby reduces the contradiction to previous findings.

The nonsignificance of the other possible predictors sheds a new light on some of the “usual suspects” [20]. In contrast to earlier research [1, 47] our data revealed no association between psychotic symptoms and medication attitudes. We found no support for the assumption that patients with more positive symptoms have a more negative opinion about medication as was found by Haq et al. [47]. In contrast to Haq et al. [47], however, we assessed symptoms via self-report. Although self-report measures of positive symptoms have been shown to be valid [48] the differences in assessment might nevertheless be responsible for the diverging findings. Insight has been shown to be the most significant predictor of patient-clinician discrepancies in positive symptoms rating with lower insight predicting higher clinician compared to patient ratings [48]. To avoid a confounding impact of insight on the association of symptoms and medication attitudes, future studies might, therefore, need to consider including both patient and observer ratings of positive symptoms.

Although the perceived quality of the relationship to the physician was correlated with medication attitudes in the bivariate analysis the association did not remain significant in the multivariate analysis. This was surprising in the light of consistent previous findings [1, 14, 17–19]. This result could be partially due to a substantial correlation between relationship ratings and insight into the need for treatment (*r* = 0.54,  *P* < 0.001). A post hoc Sobel test suggested that the association between the perceived relationship and medication attitudes is mediated by insight into need for treatment (*z* = 3.48,  *P* < 0.001). More positive contact to the physician might foster the perception of needing treatment, which in turn increases positive medication attitudes.

In our data social support was not associated with medication attitudes. Previous findings on this relationship are heterogeneous [1, 15, 18, 19], which might be explained by differences in the operationalization of social support. In contrast to our assessment of general social support, the studies who found a significant relationship between medication attitudes and social support [15, 19] used a narrow definition of it as the family being involved in the treatment. Our results suggest that people with all degrees of general social integration can have positive attitudes towards medication.

Analogous to previous research [16] we found a positive bivariate association between attitudes towards medication in the immediate social environment and patients' attitudes. However, this variable did not remain a significant predictor after controlling for other possible predictors. This might be due to a statistical and conceptual overlap between this variable and insight into need for treatment (*r* = 0.50,  *P* < 0.001). Results of a post hoc Sobel test suggest that the association between medication attitudes of the immediate social network and the patients' attitudes is mediated by insight into need for treatment (*z* = 3.27,  *P* < 0.01). Friends' and family's opinion about the medical treatment could impact on the person's perceived need for treatment in general and thereby influence medication attitudes. Thereby attitudes of the immediate social environment might form a subjective norm as described by Ajzen [49].

Finally, positive metacognition (i.e., survival beliefs) about paranoid experiences was not associated with medication attitudes. With regard to negative metacognition the bivariate analysis revealed participants who perceived their paranoia as more distressing to have more positive medication attitudes. However, this association did not remain significant in the multivariate analysis, which is likely to be due to an overlap between negative metacognitive beliefs and aspects of insight. A post hoc Sobel test indicated that the relationship between negative metacognition and medication attitudes is mediated by insight into need for treatment (*z* = 2.51,  *P* < 0.05). A more negative perception of paranoid experiences and their consequences is likely to increase the awareness of needing treatment, which in turn promotes positive medication attitudes.

### 4.2. Predictors of Adherence

It is noteworthy that our process model explained a substantial amount of the variance in medication attitudes and a significant, albeit small, proportion of the variance in adherence. This can be explained by the general discrepancy between attitudes and behavior that rarely correlate to more than *r* = 0.30 [50]. Another reason might be that we focused on evaluative factors, whereas more practical reasons for nonadherence such as forgetfulness or disorganization were not assessed and should be included in future research.

### 4.3. Strengths and Limitations

The test of the differentiated process model to explain medication attitudes and adherence addresses a central problem of earlier research and presents a key strength of our study. A further advantage is the comprehensive set of possible predictors, which we assessed systematically. Furthermore, the online assessment probably reduced the danger of biased responses. The dropout rate of 50% corresponds to the average proportion of completed surveys in the online survey system SoSci-Survey [51]. This indicates that it might have been due to the nature of online studies in general, in which distracting factors or absence of personal contact to the researcher tend to lead to reduced engagement of the participants. However, we compared completers and dropouts with regard to demographic variables and found no differences in matters of age, gender, level of education, or diagnoses. This indicates that there was no systematic dropout. Nevertheless, as our sample was not large enough for a crossvalidation, our results need to be replicated with a larger sample to achieve a more precise estimation of the model and to control for possible biases in the sample. A possible selection effect has to be considered because parts of the sample were assessed via online forums and self-help groups. Participation in these forums and groups might have influenced the attitudes. The cross-sectional design limits the conclusions that can be drawn (e.g., the participants and their immediate social environment might influence each other in both directions with regard to their attitudes towards medication). The self-reported diagnoses pose a further limitation as they were not testable. However, several aspects indicate the accuracy of the data. Firstly, there was no incentive for lying as participation in the lottery was also possible for people who had not reported a psychotic diagnosis. Secondly, regarding the proportions of diagnoses our sample is representative of patients with psychotic disorders [52] and recent research indicates that online studies are a fairly reliable way to assess psychotic symptoms [53]. Finally, although we assessed a large number of possible predictors, future research could incorporate even more differentiated constructs such as the person's confidence that the disorder can be treated or further aspects of psychopathology such as depression which has been shown to be associated with adherence in somatic disorders [54].

### 4.4. Practical Implications

The study draws the attention to an assortment of factors that should be addressed when discussing medication issues with patients. For one, it seems important to assess whether the patient perceives himself or herself to be in need of treatment in general and—if this is not the case—attempt to strengthen the patient's insight into the need for treatment. Following Zygmunt and colleagues' findings [55], it is promising to illustrate that medical, along with psychosocial, treatments could be helpful for the patient to achieve personal goals. At the same time, following West's request for patients' self-determination [9], the clinician should accept a rejection of medical treatment if a patient concludes that medication will not help to achieve personal goals. Additionally, following the results of the mediation analyses the relationship to the treating physician, the immediate social norm and negative metacognition seem to be relevant for the insight into need for treatment. Clinicians should aim at a positive and cooperative relationship to the patient in order to foster the trust into the treatment and to increase the adherence to it. Family members or friends could be included into the discussion in order to promote the benefits of possible medical or psychosocial therapies and thereby establish a common agreement on the treatment. Furthermore, clinicians might consider negative metacognition when discussing the perceived need for treatment. In the case of patients who are not aware of the negative consequences of their symptoms it could be promising to evaluate the personal costs of the symptoms together with the patient and then strengthen the confidence in the treatment to overcome these adverse consequences. In addition, our results suggest that it is beneficial for the establishment of positive medication attitudes if a patient attributes his or her symptoms to a mental disorder. Such an attribution might help to clarify the patient's problems, which is generally part of a successful therapeutic process [56]. Thereby it could strengthen the patient's motivation to adhere to all parts of a treatment plan, including medication. However, such an attribution bears the risk of consolidating a self-perception as being ill. In contrast, normalizing beliefs that describe symptoms as being common experiences are seen as functional and helpful for patients [43]. Therefore, professionals should aim at a balance between clarifying the symptoms and taking them seriously on the one hand and normalizing them on the other hand. Furthermore, our results indicate that integrating biological causal explanations into the personal model of the disorder could be helpful to improve medication attitudes. The clinician could actively strengthen biological explanations or use a Socratic dialogue to introduce them to patients who are likely to benefit from medication. However, taking into account the results by Lüllmann et al. [23], a combination between psychosocial and biological causes should be aspired. Our results also suggest that the clinician should actively assess the side effects of antipsychotic medication that have been experienced or are expected by the patient. The choice of a specific substance should aim at minimizing side effects that are likely to undermine positive medication attitudes and adherence. Furthermore, as described by Rief et al. [57], clinicians need to keep in mind that negative expectations might increase the perception of side effects. The clinician should provide information about possible side effects in a way that reduces this nocebo effect. Among other suggestions Colloca and Miller [58] recommend framing the information “positively” by focusing on the proportion of patients who do not experience the side effect. Furthermore, following Meehan et al. [59], coping strategies for side effects should be strengthened.

In order to remain open minded it can be helpful for clinicians to be aware that antipsychotic medication is not helpful for every patient [60] despite the fact that they are recommended as a standard treatment [61]. In Leucht and colleagues' meta-analysis [62] the effects of antipsychotic medication were consistent but smaller than expected (mean moderate effect size of −0.51) and responder rates were low. The authors concluded that six patients needed to be treated with a second-generation antipsychotic drug in order to achieve a significant improvement for one patient. Furthermore, long term risks are becoming more and more apparent [63, 64]. In contrast, there is encouraging support for a treatment with minimal use of antipsychotic medication (e.g., the Soteria approach [65]) and for psychological interventions, such as CBT, as an alternative to medication [66]. Therefore, if a patient decides against a medical treatment as a result of a shared and informed decision process the clinician should be open to accept this as a rational choice.

## Figures and Tables

**Table 1:** Bivariate correlations between medication attitudes, medication adherence, and possible predictors (*n* = 84).

	Attitudes	Adherence	Insight				Psychopathology		Side effects	Alliance	Social support	Causal beliefs		Metacognition	
	BMQ	MAQ^a^	SUMD^a^	FKE-SA	FKE-LS	FKE-NT	CAPE-POS^a^	CAPE-NEG	GASE, UKU	HAQ, BFTB	F-sozU K-14	IPQ-S-BIO	IPQ-S-PSY	BAPS-POS^b^	BAPS-NEG^b^
MAQ^a^	0.30**														

SUMD^a^	0.23*	0.22*													
FKE-SA	0.24*	−0.10	0.02												
FKE-LS	0.34**	0.03	0.30**	0.82***											
FKE-NT	0.53***	0.12	0.38***	0.37***	0.45***										

CAPE-POS^a^	0.08	−0.20	−0.13	0.64***	0.51***	0.19									
CAPE-NEG	0.16	−0.22*	0.07	0.43***	0.39***	0.35**	0.57***								

GASE, UKU	−0.36**	−0.20	−0.17	0.25*	0.15	−0.12	0.30**	0.00							

HAQ, BFTB	0.34**	0.00	0.23*	0.11	0.12	0.54***	0.08	0.13	−0.20						

F-sozU K-14	0.00	0.09	−0.08	0.09	0.00	0.25*	−0.08	−0.12	0.20	0.23*					

IPQ-S-BIO	0.43***	0.03	0.40***	0.26*	0.34**	0.42***	0.16	0.29**	−0.08	0.33**	0.02				
IPQ-S-PSY	−0.01	0.01	0.27*	0.23*	0.28*	0.35**	0.32**	0.17	0.10	0.14	0.00	0.22*			

BAPS-POS^b^	0.18	−0.17	0.07	0.37**	0.26*	0.07	0.53***	0.25*	0.40***	−0.01	−0.09	0.07	0.21		
BAPS-NEG^b^	0.31**	−0.03	0.24*	0.49***	0.59***	0.32**	0.48***	0.44***	0.08	0.03	−0.11	0.28*	0.09	0.25*	

MASE	0.38***	−0.03	0.08	0.23*	0.20	0.50***	0.23*	0.22*	−0.05	0.25*	0.31**	0.17	0.11	−0.05	0.23*

^a^Values are Spearman's correlations (nonparametric scales); ^b^reduced sample size due to filter question (*n* = 78); *P < 0.05; **P < 0.01; ***P < 0.001; MAQ: Medication Adherence Questionnaire; BMQ: Beliefs about Medicines Questionnaire; SUMD: Scale to Assess Unawareness of Mental Disorder; FKE: Questionnaire of Illness Insight; FKE-SA: Symptom Awareness; FKE-LS: Labeling of Symptoms; FKE-NT: Need for Treatment; CAPE: Community Assessment of Psychic Experience; CAPE-POS: Positive Symptoms; CAPE-NEG: Negative Symptoms; GASE: Generic Assessment of Side Effects; UKU-SERS: UKU Side Effect Rating Scale; HAQ: Health Alliance Questionnaire; BFTB: Bonn Questionnaire for Therapy and Consulting; F-sozU K-14: Social Support Questionnaire; IPQ-S: Illness Perception Questionnaire for Schizophrenia; IPQ-S-BIO: Biological Causal Beliefs; IPQ-S-PSY: Psychological Causal Beliefs; BAPS: Beliefs about Paranoia Scale; BAPS-POS: Positive Metacognition; BAPS-NEG: Negative Metacognition; MASE: Medication Attitudes of Social Environment.
